# Call them COVIDiots: Exploring the effects of aggressive communication style and psychological distance in the communication of COVID-19

**DOI:** 10.1177/0963662521989191

**Published:** 2021-01-30

**Authors:** Haoran Chu, Shupei Yuan, Sixiao Liu

**Affiliations:** Texas Tech University, USA; Northern Illinois University, USA; University at Buffalo, USA

**Keywords:** aggressive communication, COVID-19, expectancy violation, psychological distance

## Abstract

This study examined the influences of perceived distance to communicator on the effects of aggressive style (i.e. personal attacks and intense languages) in communicating scientific issues such as COVID-19 to the public. With a multi-site experiment (*N* = 464), we found that aggression led to a heightened violation of expected social norm regarding communication styles. However, the interpretation of violation varied depending on the individual’s perceived distance to the communicator. Close distance articulated the urgency and severity of COVID-19 risks conveyed with aggression, which further increased compliance with the message. Far distance perception amplified aggression’s negative influence on writer likeability. The findings showed that aggressive communication may generate positive outcomes when dealing with public understanding of scientific issues such as COVID-19, but communicators need to build a closer connection with their audience.

The widespread transmission and high mortality rate of Coronavirus Disease 2019 (COVID-19) pose severe challenges to public health systems across the world (World Health Organization (WHO), 2020). As of December 2020, more than 17 million cases of COVID-19 have been reported in the United States and more than 70 million cases across the world (WHO, 2020). As an effective treatment for the disease is yet to be widely available, governments and healthcare organizations worldwide are taking preventive measures to slow down the spread of the disease and avoid overloading healthcare systems (Thunström et al., 2020). However, despite the numerous calls by scientists, healthcare workers, and government officials to take actions such as avoiding social gathering or wearing protective equipment, certain segments of the public in the United States and other countries are showing strong resistance to these scientific recommendations (Brennan, 2020). Fueled by economic, political, and ideological interests, the reluctance has heated up from personal behavior to open debates online and offline.

Due to the severity of COVID-19 and the urgency of collective efforts to mitigate its impacts, the ongoing opposition frustrates scientists and informed individuals, leading to intense criticism and even name-calling. Some started to use uncivil language to criticize people who are reluctant or refuse to follow scientific recommendations in taking preventive measures against COVID-19, such as those protesting against mask wearing mandate and the stay-at-home orders. Among these criticisms, “COVIDiots” emerged as a symbolic term that crystallizes such frustration over the motivated skepticism of science in the pandemic (Mahdawi, 2020). The coinage of “COVIDiots” should not be taken as a singular incident. It reflects the long-standing disappointment in the persistent politicization of science (Bolsen and Druckman, 2015). In response to such challenges, science communicators have developed strategies such as message framing and narratives to reduce counterarguing and increase compliance with scientific recommendations (Liu and Yang, 2020a). Among them, aggressive communication is characterized with intense tones and attacks on person or persons who oppose the communicator’s view (Yuan and Lu, 2020). There has been a tradition of using aggressive communication style to demean competitors in political contexts such as campaign advertisements and rallies (Lau et al., 2007). Although science is rooted in objectivity and neutrality, the increasing politicization of scientific issues make the discussion of science less civil. Indeed, though there is no formal observation of frequencies and intensities of aggression in science communication messages, a quick keyword search of derogatory terms such as “COVIDiots” on Twitter yields thousands of tweets, reflecting the widespread use of aggressive language in this debate.

When name-calling or uncivil and aggressive utterances have become more and more common in conversations about COVID-19, we still know little about how audiences perceive these messages. Besides seeing aggressive messages as the author’s emotional expression, we also need to recognize the influences of aggressive communication on individual audience’s decision-making. This is especially true as the responsibility of communicating science and debunking widespread misinformation has been decentralized from a small group of experts to informed individuals embedded in large social networks (Tambuscio et al., 2015). Such a trend calls for further investigation into the relational dynamics between science communicators and their audience and more importantly, its impacts on public understanding of science.

In response to the challenges illustrated earlier, this study examines the effects of aggressive style in science communication based on expectancy violation theory (EVT) and construal level theory (CLT) of psychological distance. We attempt to understand how aggressive communication styles can be used to maximize the desired outcomes depending on the relational characteristics between communicators and audience. Practically, this study intends to provide implications for science communicators to offer effective advice to help individuals make decisions based on science. We focus on psychological distance’s moderating effects on people’s formation and interpretation of expectancy violation when processing aggressive messages. In the following section, we review EVT and CLT in the context of science communication and illustrate the corresponding hypotheses.

## 1. Literature review

### Aggressive communication and expectancy violation

Research on aggressive communication finds its root in interpersonal communication contexts. Some related concepts include verbal aggression, which involves attacks on the other’s self-concept (i.e. ad hominem attack) above and beyond their opinions (Infante, 1987), and incivility or swearing, which focuses on the use of intense language or even profanity in communication (Johnson, 2012; Mutz and Reeves, 2005). The different research foci also signal that aggressive communication assumes multiple defining characteristics. First, it is more intense than neutral or polite communication with the use of charged words and even expletives (Johnson, 2012). In addition, it features hostility against opponents instead of, or in addition to their opinions (Greenberg, 1976). Synthesizing these two characteristics, we define aggressive communication style *as a style of language that combines intense messages with lack of respect and an attack on a person or group* (Yuan et al., 2018).

Although aggressive communication often emerges in interpersonal interactions, its existence goes beyond the singular environment and has been studied in other contexts such as mass communication (Seiter and Gass, 2010) and organizational communication (Johnson, 2012; Myers, 2002). Recent science communication studies have also begun to recognize the use and function of aggressive style in disseminating science information such as information related to genetically modified organisms (GMO) and climate change (Yuan et al., 2019b; Yuan and Lu, 2020). Research shows that aggressive communication can sometimes generate positive effects during communication processes. For example, Mutz and Reeves (2005) found that aggressive attack of opponents enhances the entertaining an effects of political messages. In the science communication context, research shows that people favored an aggressive communicator over a polite one when communicating about GMO, but such an effect was only observed among those holding positive preexisting attitudes toward GMO (Yuan et al., 2019b). However, aggressive style does not always lead to desired outcomes. For instance, some found that aggressive campaign messages damage the trustworthiness of political candidates (Lau et al., 2007). In science communication, uncivil and intense messages also exert detrimental impacts on perceived source credibility, and more importantly, compliance with the scientific recommendations (Yuan et al., 2019a). Previous research has attempted to explain the mixed findings on the effects of aggressive communication by including other factors that may affect individuals’ perceptions, including the characteristics of the communicator such as gender or role (Yuan and Besley, 2017). However, as there is yet no conclusive explanation of aggressive communication style’s effects, further investigation is needed to address the inconsistent findings.

EVT is a viable perspective to untangle the complex interplay of communication, relational, and contextual factors in shaping the influence of aggressiveness on persuasion outcomes (Burgoon and Hale, 1988; Yuan and Lu, 2020). EVT states that people hold certain expectations of others’ behaviors in communication. Violating such expectations often leads to a shift in arousal level which subsequently results in accelerated change in attitudes and behaviors (Yuan et al., 2018). EVT centers on the assumption that there exist expectations of how people should and would communicate in different contexts, depending on the issue being communicated, the relationship between communicators, and the characteristics of communicators (Burgoon and Hale, 1988).

Although EVT was first developed to examine the violation of nonverbal norms such as physical proximity and gaze in interpersonal conversations, its utility extends to other settings. As aggressive communication often signals and reflects the deviation from a neutral and calm conversation, the aggressive message is likely to violate the expected composure and tone of a competent communicator. Such violations then, for example, negatively influence trust in the communicator and intention to follow her recommendation (Yuan and Lu, 2020). Research shows that expectancy violation effectively explains the influences of communicative aggressiveness on people’s responses to messages in various contexts (Johnson, 2012; Yuan et al., 2019b). Specifically, aggression often leads to increased expectancy violation, which negatively relates to the intended outcomes of persuasive messages. Therefore, we expect that individuals will find aggressive communication on COVID-19 in violation of their expectations and have subsequent responding actions.

*H1a*. Aggressive message on COVID-19 will lead to stronger expectancy violation.
*H1b*. Expectancy violation mediates the influence of aggressive communication style (versus neutral communication) on individual’s compliance with the message on COVID-19 prevention.

### Psychological distance perception and expectancy violation

As illustrated earlier, aggressive communication style may violate people’s expectation of a composed and calm science communicator. However, the level of perceived violation and its subsequent influences on message compliance may vary. Specifically, as various relational and individual characteristics may influence the formation and interpretation of expectancy violation, aggressive communication may lead to different persuasive outcomes depending on these factors (Burgoon and Hale, 1988). Among them, we focus on psychological distance to explicate its conditional effects of aggressive communication style and expectancy violation on public understanding of scientific issues such as COVID-19 prevention.

Psychological distance is an idiosyncratic construct that denotes the perceived distance between a target object or person and the observer at here and now (Fujita et al., 2006; Trope and Liberman, 2010). Trope and Liberman (2010) suggest that there exist four interrelated dimensions of psychological distance, including spatial distance (i.e. 1000 miles is farther away than 1 mile), social distance (i.e. a friend is closer than a stranger), temporal distance (i.e. tomorrow is closer than 1 year from now), and hypothetical distance (i.e. a sure event is often perceived closer than a low-probability event). These distance dimensions are closely associated with each other, insofar as change in one dimension often leads to change in the others. For instance, people often perceive someone living in a geographically remote location as socially distant than those living in their neighborhood.

In the current research, we operationalize perceived social distance between communicators as homophily, which denotes the perceived similarity between the observer and the target person (Rogers and Bhowmik, 1970). Homophily has long been utilized to capture social distance perception (Liviatan et al., 2008). High level of similarity is associated with close distance as people tend to perceive those similar to them as socially close (Hernández-Ortega, 2018; Liviatan et al., 2008). As illustrated earlier, as change in spatial and other distance dimensions (e.g. time difference) often leads to variation in social distance, we expect that local communicators will be perceived as socially closer and of high homophily to their audience, than a distant communicator. Change in communicator distance is especially relevant to the communication of the COVID-19 pandemic, as the public are constantly receiving information about the disease and its preventive measures from different sources (e.g. news media, local and national public health authorities) that are close or distant to them.

Another important proposition made by CLT is that psychological distance often influences people’s response to stimuli through the mediation of construal level, which denotes the mental representation of a target object (Trope and Liberman, 2010). Specifically, high-level construal is associated with abstract and context-independent perceptions, while low-level construal involves more concrete and context-specific information. As increases in distance often render the contextual details of stimuli less available to one’s mental construal, far distance often leads to more abstract and high-level construal, while short distance associates more with concrete low-level construal (Trope and Liberman, 2010). For example, when asked to describe a party that took place long ago, people often use abstract adjectives such as happy and joyful, while concrete terms such as balloons and cakes are often used to describe a recent gathering (Trope and Liberman, 2010). As close distance often increases the availability of concrete cues in low-level mental construal of objects, while far distance elaborates the influences of abstract, high-level construal, distance also determines the influences of abstract or concrete factors on people’s response to the message.

Of note, change in actual distance may not always lead to uniform change in distance perception. Different distance cues embedded in messages may generate far or close distance perception depending on people’s ability to construe the target object (e.g. the communicator in our case). For example, Chu and Yang (2019) found that trait empathy, which characterizes a person’s ability to take other people’s perspectives and feel their pain or joy (Davis, 1980), moderated distance cues’ impacts on perception. Specifically, people with lower trait empathy were more susceptible to change in distance manipulation as they may be less likely or capable of forming concrete perceptions of distant others. We therefore hypothesize that people scoring lower in trait empathy may be more influenced by distance framing in a way that they will perceive the out-of-state communicator (compared to the local communicator) as more distant than those with higher trait empathy.

*H2.* Trait empathy will moderate distance framing’s influence on distance perception insofar as the difference in distance perception caused by the far or close distance frame will be greater among people with lower trait empathy than people with higher trait empathy.

Psychological distance is not a novel concept in science communication, but most research in the field focuses on the perceived distance to issues being discussed, such as perceived distance to climate change and health threats (Chu and Yang, 2018; Liu and Yang, 2020b). What has largely been under-addressed is how the perceived distance to the message communicator influences people’s response to it. Perceived distance to the communicator is particularly relevant to our explication of aggressive style’s influences in communications about COVID-19 for a few reasons. First, psychological distance often influences the way we communicate with other people. For example, Stephan et al. (2010) found that we are often more polite to people who are socially distant to us (e.g. a stranger). Similarly, EVT research also shows that people react differently to violations conducted by friends or strangers (Burgoon and Hale, 1988). Second, as COVID-19 is an ongoing crisis that exerts severe impacts on almost everyone’s life, people’s experience with the pandemic may bias the effects of experimental messages featuring different distances to the disease. For instance, a person who was exposed to the disease may perceive it as psychological close, despite reading a message emphasizing its impacts in faraway places. Finally, as the public may receive messages on COVID-19 from communicators of different levels of distance (e.g. local vs federal public health authorities), perceived distance to the communicator is integral to the context of COVID-19 communication, and experimentally induced change in such distance should be more authentic than variation in COVID-19 distance.

Based on research in EVT and CLT, we propose that perceived distance to the communicator may influence aggressive communication’s influence through two processes. First, closer distance perception may render aggressive communication style less violating and more conforming to expectation. Second, perceived distance may influence people’s interpretation of violated expectancy, which subsequently shape their response to the message.

### Psychological distance and formation of expectancy violation

Corresponding to our first proposition, we argue that distance may influence aggressive communication’s impacts on expectancy violation for two reasons. First, close distance may increase the perceived frequency of intense and even aggressive communication (i.e. predictive expectancy; Houser, 2005). Specifically, as closer social relationships are often characterized with stronger emotional intensity (Berscheid and Ammazzalorso, 2004), experiencing an intense encounter with a friend or partner is more likely to happen than with a stranger. Supporting this argument, Jay (1992) found that swearing, an intensive and aggressive form of communication, is more prevalent among college students in social settings where they share more personal relationships, than in academic settings, where the relationships are more professional. Therefore, people may be more accustomed to intense communication style in socially close interactions and thus perceive less expectancy violation. Second, aggressive communication may be more socially acceptable at closer distance (i.e. *prescriptive* expectancy). Although not directly addressing aggressive communication, Stephan et al. (2010) found that politeness and psychological distance reciprocally influenced each other, inasmuch as far distance often leads to more polite interaction, while politeness signals and reflects social estrangement and inequality. Based on these findings, we argue that people may be more comfortable or at least tolerant of intense and even hostile interaction with individuals they feel close to.

*H3*. Aggressive communication style’s influence on expectancy violation is moderated by distance perception, in a way that the aggressive message will be considered as less violating and unexpected when the message comes from a communicator the audiences feel close to, while more violating and unexpected when it comes from a distant communicator.

### Psychological distance and interpretation of expectancy violation

Furthermore, psychological distance may also influence the way people interpret and respond to violated expectations. As noted earlier, people often respond to psychologically close or distant stimuli differently due to different levels of construal (Trope and Liberman, 2010). Low-level concrete factors often exert stronger influence when interacting with nearby objects, while high-level abstract features often weigh more in dealing with faraway issues or persons.

Among various high and low-level factors, action identification theory points to the importance of high and low-level identification of actions (Vallacher and Wegner, 1989). Specifically, the theory suggests that people may assign different meaning to behaviors, such that some are believed to originate from a person’s dispositional characteristics such as value and personality (i.e. internal attribution), while others are motivated by situational cues such as constraints and norms (i.e. external attribution). As individuals’ responses to aggressive communication and expectancy violation depend on their interpretation of such deviations from the norm, internal and external attribution is critical to our understanding of people’s response to expectancy violation. Specifically, we argue that the difference in the interpretation of expectancy violation may lead to changes in two perceptual variables that are critical to the persuasive outcomes of COVID-19 communication, namely communicator likeability and risk perception. Communicator likeability, or writer likeability in the context of written communication, refers to the audience’s attitudes toward a message writer, and favorable attitudes (i.e. high likeability) often lead to higher acceptance of persuasion (Yuan et al., 2018). As an aggressive letter may be interpreted as an expression of hostility, the message’s persuasive outcome may also be undermined due to damage to writer likeability. However, an aggressive communicator’s norm violation may also be interpreted as the outcome of frustration over the severe risks posed by the pandemic. As illustrated earlier, the politicization of scientific issues has long aggravated science communicators and led to increasingly belligerent debates. It is thus conceivable that an out-of-place message from an angry scientist or doctor (i.e. expectancy violation) may be driven by his or her recognition of the risk being communicated.

Coming back to our discussion on construal level and expectancy violation, internal attribution, due to its centrality to the perception and evaluation of a person, tends to be associated with higher construal level and far psychological distance (Fujita et al., 2006). For example, people are more likely to attribute a socially distant person’s behavior to his or her traits (e.g. stepping on one’s toe due to being clumsy), a high-level characteristic, while attributing the same behavior to contextual constraints (e.g. stepping on one’s toe because it was crowded) for a socially closer other (Trope and Liberman, 2010). In the COVID-19 context, far distance perception may prompt individuals to explain the violated expectation as “the doctor writes aggressively because he is an aggressive and hostile person,” which damages the image of the communicator and leads to reduced writer likeability. Indeed, research shows that aggressive communication style had negative impacts on writer likeability, and such effects were more salient among people whose preexisting attitudes diverge from the writer’s (Yuan et al., 2019b). Furthermore, as attitudes often shape people’s intention to engage in different behaviors, it is possible that expectancy violation caused by aggressive communication style may negatively influence people’s intention to follow the recommendation given by a distant communicator who they like less.

*H4a*. Expectancy violation interacts with distance perception to influence writer likeability in a way that perceived violation exerts stronger negative impacts on likeability when the perceived distance is far.
*H4b*. Writer likeability mediates the influence of expectancy violation and distance perception on people’s compliance with the message, where expectancy violation leads to lower intention to comply when the perceived distance is far.

Conversely, close distance often facilitates attribution of actions to situational constraints which are more accessible in concrete construal of target persons (Trope and Liberman, 2010). Therefore, when interacting with an aggressive communicator to whom audiences feel close, people may ascribe his or her violation of social norms to external factors. As illustrated earlier, one important external driver of norm-violating aggression in the COVID-19 context is the severity of the pandemic and the urgency to contain its spread (e.g. “the doctor writes aggressively because the situation is urgent”). Consequently, expectancy violation may enhance the recognition of the severe risks posed by the pandemic (i.e. risk perception) when appraised at closer distance. Research has identified risk perception as one of the major drivers of people’s intention to take preventive actions against health risks (Neuwirth et al., 2000; Slovic, 2000). It is, thus, possible that recognition of the imminent threats of COVID-19 would increase people’s compliance with the recommendations to support preventive policies and actively protect themselves from COVID-19.

*H5a*. Expectancy violation interacts with distance perception to influence risk perception in a way that perceived violation exerts stronger positive impacts on risk perception when distance is closer.
*H5b*. Risk perception mediates the influence of expectancy violation and psychological distance on people’s compliance with the message, where expectancy violation leads to stronger intention to comply when the distance is closer.

**Figure 1.**
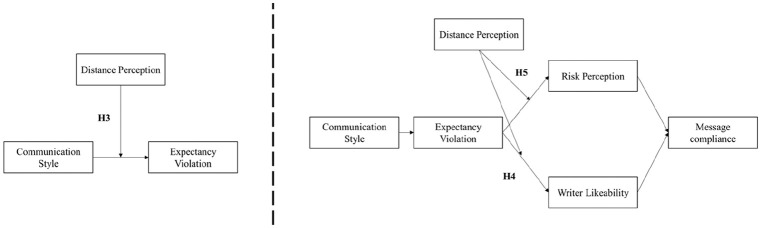
Relationships hypothesized in H3–H5.

Relationships hypothesized in H1 and H3–H5 are presented in Figure 1.

## 2. Method

### Sample and procedure

Upon approval from authors’ institutional review boards, a multi-site survey experiment was conducted in three large public universities in Texas, Illinois, and New York from 29 April to 14 May 2020. Students enrolled in communication courses in three universities were invited to participate in an online questionnaire hosted on Qualtrics and were rewarded with course credits. In total, 721 students participated in the survey. Responses from those who did not pass two attention check challenges or did not complete the questionnaire were dropped, resulting in a final sample of 464 (64.3%), consisting of 235 students from the Texas university (50.6%), 35 students from the Illinois university (7.5%), and 194 students from the university in New York State (41.9%). There was no significant difference in participant attrition across the samples (*Pearson*’*s* χ^2^(2) = 0.95, *p* = .62), and the number of respondents from each university differ mainly due to different sizes of class enrollment. The average age of our participants is 21.03 years with a standard deviation of 3.45 (*M*_TX_ = 21.24, *SD*_TX_ = 3.87; *M*_IL_ = 23.31, *SD*_IL_ = 5.16; *M*_NY_ = 20.37, *SD*_NY_ = 2.06). In sum, 303 participants identified as female (65.3%), with 181 (77%), 18 (51.4%), and 104 (53.6%) coming from the respective universities in Texas, Illinois, and New York. The majority of respondents identified as non-Hispanic White or Caucasian (*N* = 267, 57.5%; TX: *n* = 145, 61.7%; IL: *n* = 17, 48.6%; NY: *n* = 105, 54.1%), followed by those identified as Hispanic or Latinx (*N* = 75, 16.2%; TX: *n* = 56, 23.8%; IL: *n* = 7, 20.0%; NY: *n* = 12, 6.2%); Black or African Americans (*N* = 62, 13.4%; TX: *n* = 20, 8.5%; IL: *n* = 8, 22.9%; NY: *n* = 34, 17.5%); Asian, Native American, or Pacific Islander (*N* = 48, 10.3%; TX: *n* = 10, 4.3%; IL: *n* = 2, 5.7%; NY: *n* = 36, 18.6%); and other race or ethnicity (*N* = 12, 2.6%; TX: *n* = 4, 1.7%; IL: *n* = 1, 2.9%; NY: *n* = 7, 3.6%).

The survey featured a two-factor experimental design (i.e. distance and communication style). Participants were asked to read a letter from a doctor who is either in a local or out-of-state hospital, and the letter was written in either a neutral or aggressive style. A multi-site experiment was utilized to isolate the effects of psychological distance. Specially, Texan participants in the far distance condition were instructed to read a letter from a doctor from Illinois, while their counterparts in Illinois and New York read a letter from a doctor based in Texas (see sample stimuli in Supplemental material). A series of univariate ANOVAs show that participants’ state, the target state (i.e. where the doctor was based), or their interaction did not exert significant influence on the key moderators, mediators, and outcome variables. Compared with previous research which only manipulated close or far distance to one group of individuals (e.g. foreign vs domestic risks for people residing in one country), a multi-site experiment effectively eliminates the confounding influence of attributes unique to the far or close locations such as economic and cultural differences (e.g. a faraway developing country in Asia vs a nearby developed country in North America). The letters are consistent in length and content except for the adaptation for local contexts. The aggressive letters featured strong language and ad hominem attack against people disagreeing with the stay-at-home order and other approaches aiming to curb COVID-19. The neutral letters did not have these features. For example, the aggressive condition has sentences like “We will get there, as long as the human’s stupidity and selfishness don’t take us even longer,” while the neutral version rephrased it to “We will get there, as long as we all follow the instructions that are made based on science.”

## 3. Results

### Measures

#### Distance perception

Distance perception was measured with four semantic differential items adopted from the perceived homophily scale (McCroskey et al., 1975). Using 5-point scales, participants indicated if they believe the letter writer was similar to them (1 “similar to me” to 5 “different from me”), like them (1 “unlike me” to 5 “like me”), behaves like them (1 “behaves like me” to 5 “doesn’t behave like me”) and thinks like them (1 “doesn’t think like me” to 5 “thinks like me”). The scale has achieved satisfactory reliability (α = .93). After re-coding, a composite score was computed to reflect far distance perception with higher scores. On average, our participants perceived the letter writer as somewhat distant to them (*M* = 2.74, *SD* = 1.05).

#### Trait empathy

Trait empathy was measured with seven items adopted from the Interpersonal Reactivity Index (Davis, 1980). Some sample items include “before criticizing somebody, I try to imagine how I would feel if I were in their place” and “I sometimes try to understand my friends better by imagining how things look from their perspective.” Participants reported if they believe these statements described them well on a 5-point Likert-type scale (1 “does not describe me very well” to 5 “describes me very well”). The scale was reliable (α = .80). After re-coding the reverse items, a composite average was computed (*M* = 3.75, *SD* = 0.69). Higher scores indicate higher level of trait empathy.

#### Expectancy violation

Expectancy violation was measured with four items (Burgoon and Walther, 1990; Yuan et al., 2019b). Participants provided responses to four items on a 5-point Likert-type scale (1 “strongly disagree” to 5 “strongly agree”). Items include “the author’s writing was appropriate as a doctor,” “the author wrote in a way I would expect most doctors to write,” “the author used a normal writing style for a doctor,” and “the author’s writing was unusual for a doctor.” After re-coding, a composite variable was created. Higher value of the variable indicates stronger perceived violation of expected communication style (*M* = 2.96, *SD* = 1.00, α = .88).

#### Risk perception

Risk perception was measured as a product term of two composite variables, perceived severity and susceptibility of risks associated with COVID-19 (Rimal and Real, 2003). Perceived severity was measured with three items including “I believe that COVID-19 is extremely harmful,” “I believe that COVID-19 has serious negative consequences,” and “I believe that COVID-19 is a severe health problem.” Responses were recorded with a 5-point scale (1 “strongly disagree” to 5 “strongly agree”), and a composite average was calculated (*M* = 4.38, *SD* = 0.74, α = .83). Perceived susceptibility was also measured with three items, which are “it is likely that I will get COVID-19,” “it is possible that I will get COVID-19,” and “I am at risk for getting COVID-19.” The same 5-point scale was used and on average our participants perceived themselves to be somewhat susceptible to the risks associated with COVID-19 (*M* = 3.13, *SD* = 0.95, α = .78). Risk perception was subsequently calculated as the product term of these two variables (*M* = 13.84, *SD* = 5.14).

#### Writer likeability

We used four semantic differential items adopted from earlier research to measure writer likeability (Short et al., 1976; Yuan et al., 2019a). With a 5-point scale, participants rated the extent to which they think the writer is sensitive (1 “insensitive” to 5 “sensitive”), warm (1 “cold” to 5 “warm”), personal (1 “impersonal” to 5 “personal”), and sociable (1 “unsociable” to 5 “sociable”). The scale achieved satisfactory fit (α = .82), and on average, our participants felt that the writer was a moderately likeable person (*M* = 3.31, *SD* = 0.93).

#### Compliance with recommendation

Message compliance was measured with two outcome variables. We first measured participants’ support for three policies relevant to the stay-at-home order or lockdown on a 5-point scale (1 “strongly oppose” to 5 “strongly support”). These policies are “stay-at-home order issued by state governments,” “temporarily ban gathering of more than 5 people who do not live together,” and “temporarily close non-essential business such as cinemas and theaters.” The items were reliable (α = .88), and on average, our participants supported these measures (*M* = 4.28, *SD* = 0.96).

Furthermore, we asked about their intention to follow the behaviors recommended in the letters. These behaviors include “stay at least 6 feet apart from people you do not live with,” “stay at home as much as possible,” “wash hands regularly with soap and water for at least 20 seconds,” “cover mouth and nose with face mask when around others,” and “clean and disinfect frequently touched surfaces (e.g. doorknob, desk) daily.” Participants indicated if they were likely to perform these behaviors on a 5-point scale (1 “extremely unlikely” to 5 “extremely likely”). The scale was reliable (α = .73), and on average our participants indicated strong intention to follow the recommendations (*M* = 4.46, *SD* = 0.61).

### Hypotheses testing

In response to H1a (communication style’s direct effect on expectancy violation), a regression model was analyzed. Results indicate that the aggressively toned message led to higher level of expectancy violation (*B* = 0.77, *SE* = 0.09, *p* < .001). Another regression model was employed to test the effects of distance framing on distance perception moderated by trait empathy (H2). As expected, distance framing and trait empathy interacted to predict distance perception (*B* = –0.30, *SE* = 0.14, *p* = .036). Specifically, reading a message from an out-of-state doctor led to more distal social perception only among individuals with lower trait empathy (conditional effect of distance framing when trait empathy is at one standard deviation below the mean: *B* = 0.36, *SE* = 0.14, *p* = .012). The Johnson-Neyman method was utilized to probe the significant region of this moderation. We found that only among people with comparatively lower trait empathy (lower 36.9% of the sample) did the close distance frame lead to a significant decrease in distance perception.

We utilized the PROCESS macro in IBM SPSS 26 to analyze if expectancy violation mediated aggressive communication’s impacts on people’s intention to follow the message recommendation (H1b). Expectancy violation was not directly related to policy support (*B* = –0.07, *SE* = 0.05, *p* = .13) or behavioral intention (*B* = –0.04, *SE* = 0.03, *p* = .17). However, it is notable that aggressive communication was directly associated with stronger policy support (*B* = 0.20, *SE* = 0.10, *p* = .04) and behavioral intention (*B* = 0.14, *SE* = 0.06, *p* = .02), suggesting that an aggressive message may increase compliance. Expectancy violation did not mediate the effects of communication style on policy support (*B* = –0.06, *SE* = 0.04, 95% CI = [–0.13, 0.02]) or behavioral intention (*B* = –0.03, *SE* = 0.02, 95% CI = [–0.08, 0.01]), rejecting H1b.

H3 hypothesizes that distance perception moderates aggressive communication’s impacts on expectancy violation. We tested this possibility with two regression models respectively including distance framing and perception as moderators of communication style’s impacts on expectancy violation. Results of the regression models show that aggressive communication style led to stronger expectancy violation (*B* = 0.77, *SE* = 0.12, *p* < .001) than the neutral style regardless of distance frames or perception (*B*_Communication Style × Distance Framing_ = 0.01, *SE* = 0.17, *p* = .96; *B*_Communication Style × Distance Perception_ = 0.10, *SE* = 0.08, *p* = .21). H3 was thus not supported. Notably, distance perception was positively related to perceived expectancy violation (*B* = 0.22, *SE* = 0.06, *p* < .001), indicating that the less similar to them our participants perceive the letter writer to be, the more likely they considered the writer’s tone a violation of social norms.

**Figure 2.**
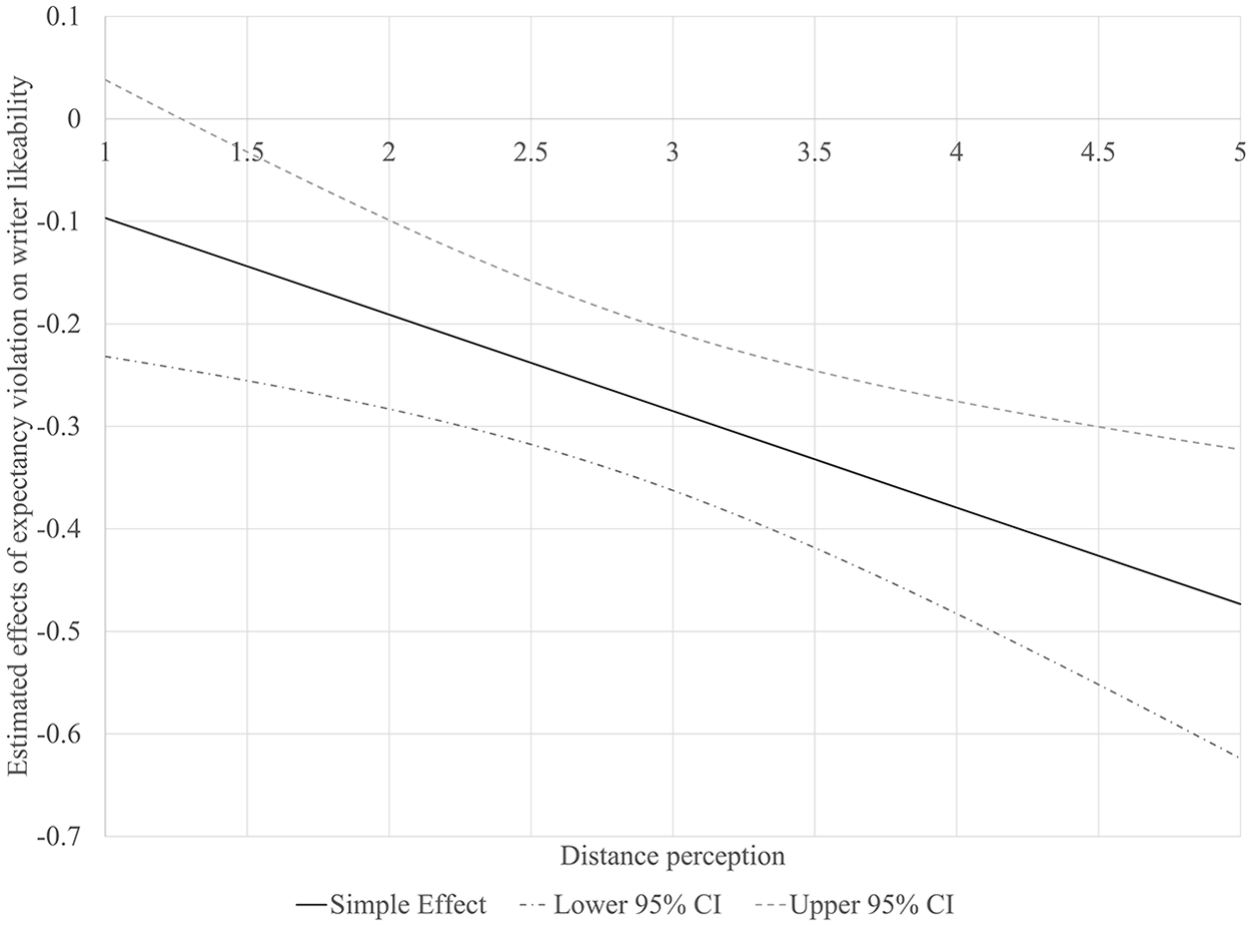
Significance region of expectancy violation’s effects on writer likeability moderated by distance perception. The simple effect is significantly different from 0 when its 95% confidence interval does not cross the horizontal axis (0).

**Figure 3.**
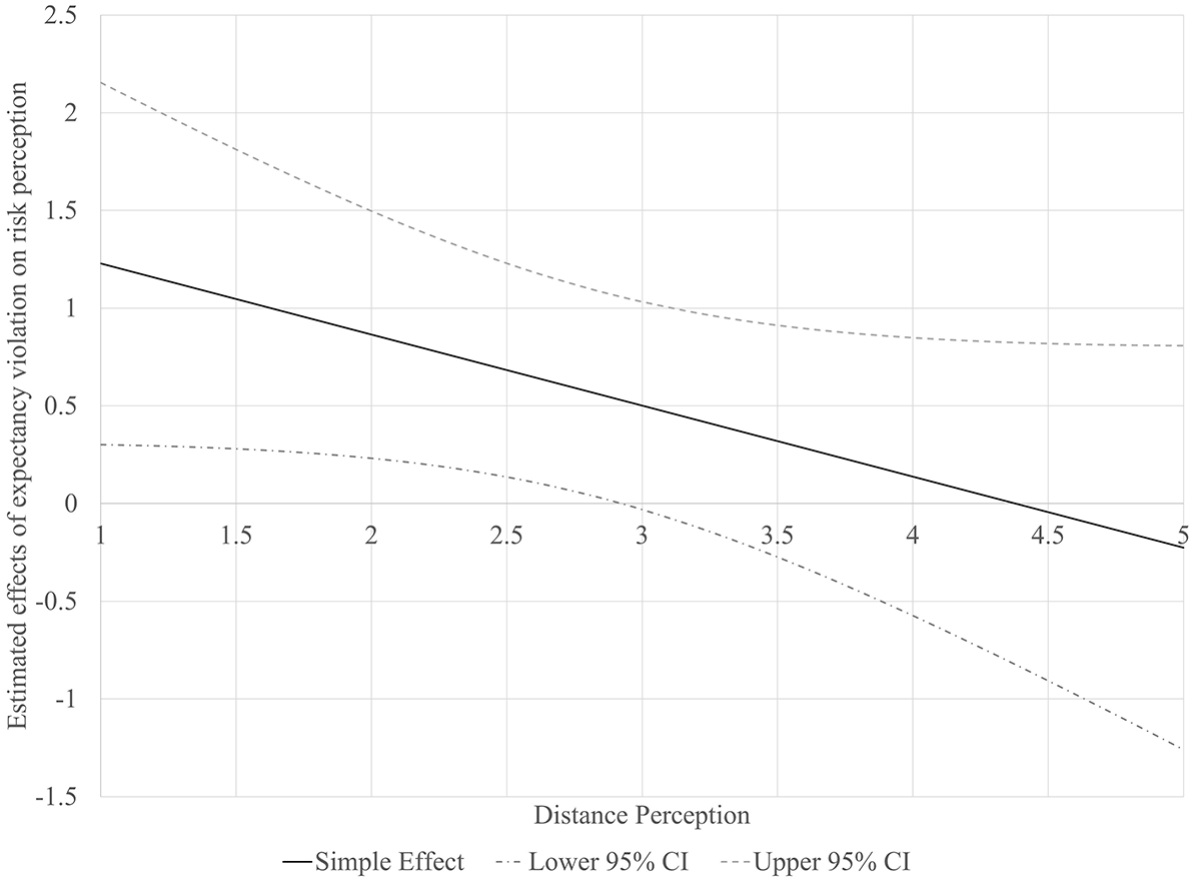
Significance region of expectancy violation’s effects on risk perception moderated by distance perception. The simple effect is significantly different from 0 when its 95% confidence interval does not cross the horizontal axis (0).

The last set of hypotheses (H4 and H5) tested if distance perception influences the attribution of aggressiveness to either the writer’s character or the urgency of COVID-19. Two customized moderated mediation models were analyzed using the PROCESS Macro (Hayes, 2017). A dummy-coded communication style variable (1 “aggressive letter,” and 0 “neutral letter”) was utilized as the independent variable, while expectancy violation, writer likeability, and risk perception were utilized as the mediators of its impacts on policy support or behavioral intention. Results from the regression models are presented in Table 1 and the conditional indirect effects of communication style on policy support and behavioral intention are reported in Table 2. Distance perception moderated the influence of expectancy violation on writer likeability. A closer inspection of the significance region of expectancy violation’s simple effects on writer likeability (Figure 2) shows that expectancy violation’s negative association with likeability grows along with distance perception. However, the interaction between expectancy violation and psychological distance was not significantly related to risk perception (*B* = –0.36, *SE* = 0.21, *p* = .08). However, by probing into the simple effects of expectancy violation conditioned by different levels of distance perception (Figure 3), we found that violation led to significantly stronger risk perception among participants who perceived the letter writer as closer to them (scoring below 51.9 percentile in distance perception). Therefore, we conclude that parts of H4a and H5a were supported.

**Table 1.** Regression models predicting writer likeability, risk perception, policy support, and behavioral intention.

	Writer likeability	Risk perception	Policy support	Behavioral intention
Intercept	3.95 (0.42)	11.23 (2.86)	4.27 (0.54)	4.35 (0.36)
Communication style^1^	−0.19* (0.07)	−0.06 (0.51)	0.14 (0.09)	**0.10**^+^ (0.06)
Distance framing^2^	0.21 (0.39)	−1.70 (2.66)	−0.90* (0.45)	−0.37 (0.3)
Trait empathy	0.13^+^ (0.07)	0.12 (0.5)	0.07 (0.08)	0.12* (0.06)
Distance framing × trait empathy	−0.07 (0.10)	0.45 (0.7)	0.25* (0.12)	0.10 (0.08)
Expectancy violation	0.00 (0.10)	1.59* (0.65)	−0.10 (0.11)	−0.10 (0.07)
Distance perception	−0.07 (0.10)	0.20 (0.69)	−0.37** (0.12)	−0.22** (0.08)
Expectancy violation × Distance perception	−0.09** (0.03)	**–0.36**^+^ (0.21)	0.04 (0.04)	0.03 (0.02)
Writer likeability			−0.02 (0.05)	−0.03 (0.04)
Risk perception			0.05*** (0.01)	0.02*** (0.01)
*R* ^2^	0.38	0.05	0.22	0.15
Adjusted *R*^2^	0.37	0.03	0.20	0.13

* *p* < .05; **p < .01; ***p < .001; ^+^*p* < .10. 1 dummy-coded, 1 = aggressive style, 0 = neutral style; 2 dummy-coded, 1 = far distance, 0 = short distance; outcome variable for each regression models are specified in the header row.

**Table 2.** Indirect effects of communication style on the outcome variables conditioned by distance perception.

Moderator value	*B* (*SE*)	Lower 95% CI	Upper 95% CI
Communication style > expectancy violation > writer likeability > policy support			
Short distance perception^a^	0.003 (0.008)	−0.012	0.019
Moderate distance perception^b^	0.004 (0.011)	−0.018	0.027
Far distance perception^c^	0.006 (0.016)	−0.025	0.036
Communication style > expectancy violation > risk perception > policy support			
Short distance perception^a^	**0.039 (0.017)**	0.010	0.076
Moderate distance perception^b^	**0.024 (0.012)**	0.002	0.051
Far distance perception^c^	0.009 (0.014)	−0.018	0.037
Communication style > expectancy violation > writer likeability > behavioral intention			
Short distance perception^a^	0.004 (0.005)	−0.005	0.017
Moderate distance perception^b^	0.007 (0.008)	−0.007	0.024
Far distance perception^c^	0.009 (0.01)	−0.009	0.032
Communication style > expectancy violation > risk perception > behavioral intention			
Short distance perception^a^	**0.018 (0.009)**	0.004	0.037
Moderate distance perception^b^	**0.011 (0.006)**	0.001	0.025
Far distance perception^c^	0.004 (0.007)	−0.008	0.019

a Conditional indirect effect of communication style when distance perception is at 1 *SD* below the mean.

b Conditional indirect effect of communication style when distance perception is at the mean.

c Conditional indirect effect of communication style when distance perception is at 1 *SD* above the mean; Significant indirect effects are bold.

Regarding the moderated mediation paths, we found that aggressive communication style (compared with neutral style) led to higher policy support and behavioral intention for COVID-19 prevention through the serial mediation of expectancy violation and risk perception. That is, emotionally aggressive messages about COVID-19 violated people’s perceived norm of how a doctor communicates, but this violation also heightened the perceived severity and urgency of COVID-19 threats, which led to increased compliance with the scientific recommendations. However, it is also notable that such effects only deviated significantly from zero when the letter writer was perceived as a socially close person. No significant mediation was found for writer likeability, as liking or disliking the writer did not significantly influence people’s compliance with the message. Therefore, H4b was not supported, but H5b was supported.

## 4. Discussion

Despite the severe and urgent damage posed by COVID-19 to societies across the world, certain segments of the public have resisted complying with scientific measures aiming to curb the threat of the pandemic. Although past studies have investigated the use of aggressive or uncivil languages across various topics (Lau et al., 2007; Mutz and Reeves, 2005; Yuan and Lu, 2020), the severity and duration of this pandemic present unique challenges for communicators advising individuals on their decision making. In response to such motivated skepticism of science, we analyzed if communicating aggressively about COVID-19 and its mitigation measures influenced people’s acceptance of scientific recommendations.

Consistent with existing research on aggressive communication and expectancy violation (Johnson, 2012; Yuan and Lu, 2020), we found that intense language and ad hominem attacks led to increased expectancy violation. However, unlike earlier research, the perceived violation did not lead to significant linear change in people’s compliance with the message. Psychological distance was introduced as a moderator of communication style and expectancy violation’s impacts on the outcome variables. Notably, our operationalization of psychological distance distinguished itself from earlier research in the context of science communication as we focused on audience distance to the communicator instead of the issue (Chu and Yang, 2019; Spence et al., 2012). As expected, distance cues embedded in experimental messages influenced distance perception of the letter writer among people with lower trait empathy (Chu and Yang, 2019), pointing to individual differences in the formation of distance perception.

We hypothesized that aggressive communication may be more acceptable and tolerable at close distance as predictive and prescriptive expectancies are influenced by the relational distance between communicators. However, distance framing or perception did not moderate communication style’s effects on expectancy violation. One possible explanation is that though intense interaction may be more frequent and acceptable between socially close communicators, the attack aspect of aggressive communication style may still be perceived as a violation of norms. Future research may consider separating different message factors of aggressive communication style and examine their interactive effects with distance perception. We may have failed to identify any interaction between aggressive communication style and distance perception due to limited sample size. As interaction effects usually require greater power to detect, further testing of the hypotheses with larger and more diverse samples may better identify the interaction between aggressive communication and distance perception.

As expected, we found that psychological distance influenced people’s interpretation of expectancy violation. Specifically, far distance led participants to interpret violation as a signal of low writer likeability. In contrary, close distance which promotes consideration of situational details, led participants to interpret violation as the writer’s anxious call for attention to the severe and imminent threats of COVID-19. Increase in risk perception also enhanced the letter’s persuasiveness, leading to stronger support for COVID-19 prevention approaches such as social distancing and lockdown, as well as intention to practice disease prevention behaviors. These findings shed light on our understanding of communication style and psychological distance. Although aggressive communication may violate the audience’s expectation of a science communicator such as scientists, doctors, and public servants, it also has the potential to highlight the urgency of the situation, when used cautiously. However, such effects depend on the relational characteristics between the communicator and audience, as feeling far or close to the communicator shapes the way audience interpret his or her intention in using aggressive language.

In conclusion, our results showed that distance to communicators may not affect perceived expectancy violation, but it plays a role in influencing the effects of violation. The possible reason is that expectancy violation is an immediate reaction (feeling surprised) while perception of the distance of the communicator requires more cognitive processing. These findings point to the importance of the strategic consideration of different communication styles which takes the relational dynamics between science communicators and the public into account. Specifically, aggressive communication styles may signal the urgency of issues being communicated and increase compliance, but such effects may only emerge when the distance between the communicator and his or her audience is close. In contrary, charged language and personal attacks may backfire when the communicator and the audience are socially distant to each other.

This study is not without limitation. Notably, we did not measure attribution of why the writer wrote aggressively. Instead, the product of internal and external attribution (i.e. writer likeability and risk perception) was utilized as the mediating factors between violation and compliance with the message. We recommend future research to include a direct measure of attribution to further validate the model proposed here. In addition, though the use of student samples made our location-based multi-site experiment possible, it may also limit the ecological validity of our findings. Particularly as demographic and psychological characteristics such as age, sex, ethnicity, and political affiliation may influence people’s reaction to and interpretation of aggressive messages, a more representative sample may allow us to explore the effects of communication style, expectancy violation, and psychological distance in different science communication contexts. Of note, we recognize that the experimentally tested relationships are less susceptible to the influence of sample characteristics, but further examination of the hypotheses in a more diverse population is warranted. In a similar vein, more diverse and ecologically valid operationalization of distance between the communicator and audience may also help pinpoint the interactive effects among psychological distance, communication style, and expectancy violation. Future research may consider manipulating distance by altering the demographics of the communicator (e.g. expert vs peer). Finally, polite communication style was not examined in this study. Due to its unique influence in science communication (Yuan et al., 2019b) and association with psychological distance (Stephan et al., 2010), we recommend future research to comparatively examine how polite, aggressive, and neutral communication style interact with psychological distance to influence the outcome of science communication messages.

## 5. Conclusion

Charged language and attack on opponents may not be what the public expect from scientists and doctors when talking about crises such as the ongoing COVID-19 pandemic. However, if used cautiously, aggressive communication style can lead to positive response from the audience, especially when they perceive the communicator as someone closer to them. Findings from this study shed light on our understanding of aggressiveness in science communication and showed that its effects are contingent on the perceived distance of the communicator. This study also has practical value. Specifically, it points to the flexibility and limitation for communicators in choosing communication styles depending on their relational characteristics with the audience. Specifically, our findings highlight the importance of building rapport and reducing distance between science communicators and their audiences. As politicization and polarization are on the rise in various aspects of our society, solidarity among scientists, communication practitioners, and the public is necessary for informed public opinion and decision-making, especially in times of crises.

## Supplemental Material

sj-pdf-1-pus-10.1177_0963662521989191 – Supplemental material for Call them COVIDiots: Exploring the effects of aggressive communication style and psychological distance in the communication of COVID-19 Click here for additional data file. Supplemental material, sj-pdf-1-pus-10.1177_0963662521989191 for Call them COVIDiots: Exploring the effects of aggressive communication style and psychological distance in the communication of COVID-19 by Haoran Chu, Shupei Yuan and Sixiao Liu in Public Understanding of Science
