# Supplementary material for: Call them COVIDiots: Exploring the effects of aggressive communication style and psychological distance in the communication of COVID-19
Source: Public Underst Sci. 2021 Jan 30;30(3):240–57. doi: 10.1177/0963662521989191 (PMC7862919; doi:10.1177/0963662521989191)
Supplement: sj-pdf-1-pus-10.1177_0963662521989191 – Supplemental material for Call them COVIDiots: Exploring the effects of aggressive communication style and psychological distance in the communication of COVID-19 [file sj-pdf-1-pus-10.1177_0963662521989191.pdf]

SUPPLEMENTAL MATERIAL

Call them COVIDiots: Exploring the effects of aggressive communication style and psychological distance in the communication of COVID-19

Haoran Chu, Shupei Yuan, Sixiao Liu

List of Content

S1. Stimulus letter for the Texas – Neutral Condition 2

S2. Stimulus letter for the New York– Aggressive Condition 3

S3. Stimulus letter for the Illinois –Neutral Condition 4

Supplemental Material

S1. Stimulus letter for the Texas – Neutral Condition (distant for participants in Illinois and New York; close for participants in Texas).

I'm a doctor at University Medical Center. Here's what I'm telling you about COVID-19.

**This message is written by Dr. John Anderson (PhD, MD) from University Medical Center in Texas. Currently, the number of confirmed cases in Texas is 20,196. Dr. Anderson is one of the health professionals fighting in the front line in Texas. This email is shared with permission from Dr. Anderson.*

Dear friends and families,

The COVID-19 pandemic is a challenge to our community unlike any we have experienced in our lifetime. For the last several weeks, I have been working nonstop for days with my colleagues, and other frontline workers. I would like to spend some time and talk about this with you.
If anyone hears from family or friends who still think this is "no big deal," or that the USA's response has been excessive, please know that they are WRONG. I'm sure you have all read about the many reasons that this is NOT "just like flu." If the rapidly growing number has not changed your mind yet, please go ahead and talk to anyone you know who works in hospitals and ask them what their situation is.

In the state of Texas, we had shelter in place order since April 2. This is not vacation time, so please understand why we cannot go to the park or lake front right now. If we fail to flatten the curve, there will be serious consequences: More people, including some of our friends and family, will get COVID-19 and even die. Healthcare workers including myself are at higher risk of getting the disease. All healthcare workers will have to witness the needless deaths of patients who could have survived. Our country is in crisis, we need to be clear about this unfortunate fact.

I know, you probably have seen the ongoing debate on when we should reopen. What's in the news these days? People who disagree with the shelter-in-place order go on the streets, gather in front of city halls and hospitals. This is a situation we are facing. Governors don't want to see their state economies struggle one moment longer than is necessary. But it is necessary for the moment. Politicians should care about the safety of their citizens and make decisions with support from data and experts. This decision should be driven by science, instead of politics.
As Americans, we all have a responsibility to do our part to reduce the spread of COVID-19 and literally help save lives. Please just do the right thing: stay at home, wash your hand, only go out if it is necessary. **We will get there, as long as we all follow the instructions that are made based on science.**

Take care and stay safe.

Best,
Dr. Anderson

S2. Stimulus letter for the New York– Aggressive Condition (distant for participants in Texas; close for participants in New York).

I'm a doctor at Buffalo General. Here's what I'm telling you about COVID-19.

**This message is written by Dr. John Anderson (PhD, MD) from Buffalo General in New York. Currently, the number of confirmed cases in New York is 277,605. Dr. Anderson is one of the health professionals fighting in the front line in New York. This email is shared with permission from Dr. Anderson.*

Dear friends and families,

The COVID-19 pandemic is a challenge to our community unlike any we have experienced in our lifetime. For the last several weeks, I have been working nonstop for days with my colleagues, and other frontline workers. I would like to spend some time and talk about this with you.
If anyone hears from family or friends who still think this is "no big deal," or that the USA's response has been excessive, please know that they are blind, or just STUPID. I'm sure you have all read about the many reasons that this is NOT "just like flu." If the rapidly growing number has not changed your mind yet, just open your eyes, go ahead and talk to anyone you know who works in hospitals, and ask them what their situation is.

In the state of New York, we had shelter in place order since March 21. This isn't some kind of vacation time, and please don't be the dumb one whining about why we cannot go to the park or lake front anymore. If we fail to flatten the curve, there will be serious consequences: More people, including some of our friends and family, will get COVID-19 and even die. Healthcare workers including myself are at higher risk of getting the disease. All healthcare workers will have to witness the needless deaths of patients who could have survived. Our country is in crisis! we need to be clear about this damning fact!

I know, you probably have seen the ongoing debate on when we should reopen. What's in the news these days? Reckless protesters take over streets, gather in front of city halls, even in front of hospitals! This is deeply troubling. Governors don't want to see their state economies struggle one moment longer than is necessary. But it is necessary for the moment. Any politician who still thinks this is a political thing, and makes decisions without support from data or experts, are irresponsibly putting their residents' lives at risk. Let science do the talk! Anyone who think they can play around science is just ignorant, or simply dumb.

As Americans, we all have a responsibility to do our part to reduce the spread of COVID-19 and literally help save lives. Just do the right thing, don't be stupid: stay at home, wash your hand, only go out if it is necessary. **We will get there, as long as the human's stupidity and selfishness don't take us even longer.**

Take care and stay safe.

Best,
Dr. Anderson

S3. Stimulus letter for the Illinois –Neutral Condition (distant for participants in Texas; close for participants in Illinois).

I'm a doctor at Northwestern Memorial Hospital. Here's what I'm telling you about COVID-19.

**This message is written by Dr. John Anderson (PhD, MD) from Northwestern Memorial Hospital in Illinois. Currently, the number of confirmed cases in Illinois is 33,059. Dr. Anderson is one of the health professionals fighting in the front line in Illinois. This email is shared with permission from Dr. Anderson.*

Dear friends and families,

The COVID-19 pandemic is a challenge to our community unlike any we have experienced in our lifetime. For the last several weeks, I have been working nonstop for days with my colleagues, and other frontline workers. I would like to spend some time and talk about this with you.
If anyone hears from family or friends who still think this is "no big deal," or that the USA's response has been excessive, please know that they are WRONG. I'm sure you have all read about the many reasons that this is NOT "just like flu." If the rapidly growing number has not changed your mind yet, please go ahead and talk to anyone you know who works in hospitals, and ask them what their situation is.

In the state of Illinois, we had shelter in place order since March 20. This is not vacation time, so please understand why we cannot go to the park or lake front right now. If we fail to flatten the curve, there will be serious consequences: More people, including some of our friends and family, will get COVID-19 and even die. Healthcare workers including myself are at higher risk of getting the disease. All healthcare workers will have to witness the needless deaths of patients who could have survived. Our country is in crisis, we need to be clear about this unfortunate fact.

I know, you probably have seen the ongoing debate on when we should reopen. What's in the news these days? People who disagree with the shelter-in-place order go on the streets, gather in front of city halls and hospitals. This is a situation we are facing. Governors don't want to see their state economies struggle one moment longer than is necessary. But it is necessary for the moment. Politicians should care about the safety of their citizens and make decisions with support from data and experts. This decision should be driven by science, instead of politics.
As Americans, we all have a responsibility to do our part to reduce the spread of COVID-19 and literally help save lives. Please just do the right thing: stay at home, wash your hand, only go out if it is necessary. **We will get there, as long as we all follow the instructions that are made based on science.**

Take care and stay safe.

Best,
Dr. Anderson
